# Duplication and independent selection of cell-wall invertase genes *GIF1 *and *OsCIN1 *during rice evolution and domestication

**DOI:** 10.1186/1471-2148-10-108

**Published:** 2010-04-23

**Authors:** Ertao Wang, Xun Xu, Lin Zhang, Hong Zhang, Lin Lin, Qin Wang, Qun Li, Song Ge, Bao-Rong Lu, Wen Wang, Zuhua He

**Affiliations:** 1 National Laboratory of Plant Molecular Genetics, Institute of Plant Physiology and Ecology, Shanghai Institutes for Biological Sciences, Chinese Academy of Sciences, Shanghai 200032, China; 2 Kunming Institute of Zoology, Chinese Academy of Sciences, Kunming 650223, China; 3 Institute of Botany, Chinese Academy of Sciences, Beijing 100093, China; 4 School of Life Sciences, Fudan University, Shanghai 200433, China

## Abstract

**Background:**

Various evolutionary models have been proposed to interpret the fate of paralogous duplicates, which provides substrates on which evolution selection could act. In particular, domestication, as a special selection, has played important role in crop cultivation with divergence of many genes controlling important agronomic traits. Recent studies have indicated that a pair of duplicate genes was often sub-functionalized from their ancestral functions held by the parental genes. We previously demonstrated that the rice cell-wall invertase (CWI) gene *GIF1 *that plays an important role in the grain-filling process was most likely subjected to domestication selection in the promoter region. Here, we report that *GIF1 *and another CWI gene *OsCIN1 *constitute a pair of duplicate genes with differentiated expression and function through independent selection.

**Results:**

Through synteny analysis, we show that *GIF1 *and another cell-wall invertase gene *OsCIN1 *were paralogues derived from a segmental duplication originated during genome duplication of grasses. Results based on analyses of population genetics and gene phylogenetic tree of 25 cultivars and 25 wild rice sequences demonstrated that *OsCIN1 *was also artificially selected during rice domestication with a fixed mutation in the coding region, in contrast to *GIF1 *that was selected in the promoter region. *GIF1 *and *OsCIN1 *have evolved into different expression patterns and probable different kinetics parameters of enzymatic activity with the latter displaying less enzymatic activity. Overexpression of *GIF1 *and *OsCIN1 *also resulted in different phenotypes, suggesting that OsCIN1 might regulate other unrecognized biological process.

**Conclusion:**

How gene duplication and divergence contribute to genetic novelty and morphological adaptation has been an interesting issue to geneticists and biologists. Our discovery that the duplicated pair of *GIF1 *and *OsCIN1 *has experienced sub-functionalization implies that selection could act independently on each duplicate towards different functional specificity, which provides a vivid example for evolution of genetic novelties in a model crop. Our results also further support the established hypothesis that gene duplication with sub-functionalization could be one solution for genetic adaptive conflict.

## Background

Gene duplication has long been recognized to be an important way to provide a substrate on which evolution acts. The classical models that predict the most possible fate of one of the duplicate genes is to degenerate to a pseudogene or get lost from the genome due to vagaries of chromosomal remodeling, locus deletion or point mutation [1-5]. A less frequent fate of the duplicate genes is to gain a new function (neo-functionalization) when the other copy still maintains its original function. However, recent studies have indicated that the newly duplicated genes are often sub-functionalized from their ancestral functions held by the parental genes [6-8]. The sub-functionalization model (also referred to as duplication-degeneration-complementation model) explains that the duplicate genes are maintained in the genome relying on complementary degenerative changes in a pair of duplicate genes, such that the duplicate genes together retain the original functions of their single ancestor [1-5,9]. During this process, the expression domain shifting is the most common character of duplicate genes. As a consequence, the duplicates acquired sub-functionalization and then were less constrained by selection than the single ancestor, which had to maintain the capacity to fulfill all functions. Therefore, selection could act independently on each duplicate and increase the gene function specificity [10].

Sequence variation plays an essential role in functional renovation of genes, however, the relationship between DNA variation and functional consequence has been enigmatic for the vast majority of genes in plant and animal kingdoms, despite an increasing number of studies have been reported. Crop species and their wild relatives with available genome information are becoming fascinating subjects for study of correlation between cryptic genetic variation and functional evolution, because they have undergone rapid diversification under intense artificial selection [11-14]. Therefore, investigating crop domestication genes will shed meaningful light on genetic variation that drives cultivation adaptation [15]. Rice was used by human about 11,000 years ago [16,17]. It has been indicated that the divergence of *indica *and  *japonica *predated rice domestication, suggesting that at least two genetically distinct gene pools of *O. rufipogon *were cultivated and subsequently domesticated [18-20]. During the long-term cultivation and domestication, tremendous diversity in rice has been selected by human, adapting to various ecosystems and agricultural management, in addition to high yielding characteristics, such as grain number and weight [12,17].

Various evolutionary models have been proposed to interpret the fate of paralogous duplicates, but little is known about the mechanisms of evolutionary change in duplicate genes leading to functional novelty. Rice has been recognized as a cereal model for such a study, and recent studies have discovered that some rice genes have undergone adaptive evolution under domestication selection [17,21]. We previously reported that the rice grain-filling gene *GIF1 *(*OsCIN2*) encoding a cell wall invertase (CWI) was most likely subjected to domestication selection [22]. Here, we report that *GIF1 *and another CWI gene *OsCIN1 *constitute a pair of duplicate genes with differentiated expression and function. Population genetic analysis showed that the two genes have experienced strong domestication selection, and interestingly, the target of selection in the *GIF1 *gene is the promoter region and that in *OsCIN1 *is the coding region.

## Results

### Evolution of *GIF1* and *OsCIN1* by gene duplication

**Figure 1:**
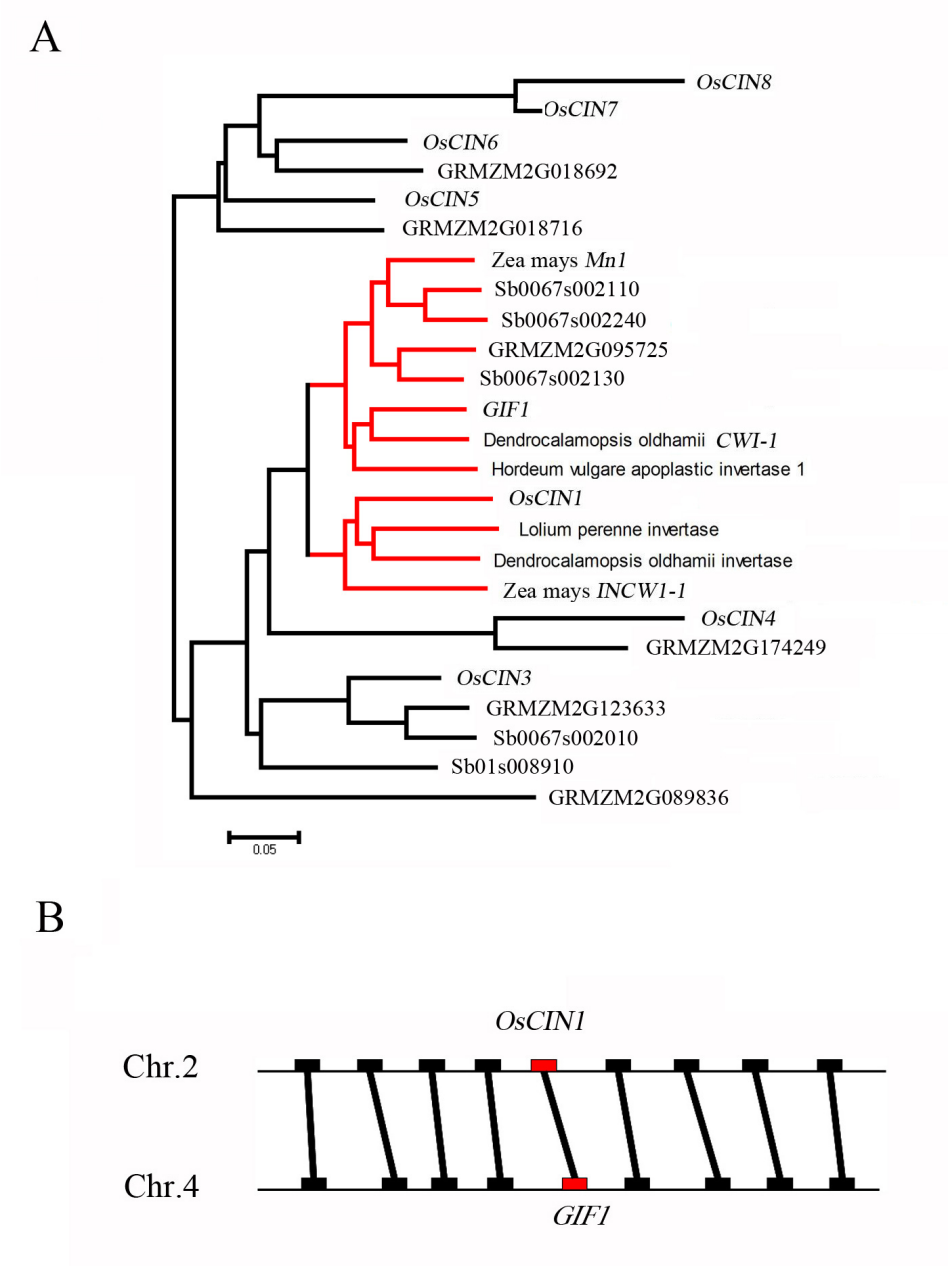
**Phylogenetic relationship of cell wall invertases and synteny of the *GIF1 *and *OsCIN1 *loci**. **(A) **The N-J phylogenetic tree constructed by MEGA program based on alignment of the DNA sequences of the 8 CWI genes of rice and CWI genes in other species, *Lolium perenne*, *Hordeum vulgare*, *Dendrocalamopsis oldhamii *and the recently released maize and sorghum genomes. Note that the rice *GIF1 *and *OsCIN1 *genes were paralogous within two subgroups. **(B) **Synteny between the *GIF1 *and *OsCIN1 *genome regions is illustrated schematically with homologous genes, indicating their duplication event.

Rice genome has a CWI family consisting of eight members [23,24]. Our previous study has demonstrated that *GIF1 *is a member of the gene family and required for assimilated carbon partitioning during early grain-filling [22]. A phylogenetic analysis of the known plant CWI genes and predicted CWI genes from the recently released maize and sorghum genomes showed that *OsCIN1*, located on chromosome 2, is highly similar to *GIF1 *located on chromosome 4 (Figure 1A). Genetic distance based on amino acid substitutions also indicated that OsCIN1 is most closely related to GIF1 (Additional file 1). To gain insight into their evolutionary relationship, the 500-kb flanking sequences of the *GIF1 *and *OsCIN1 *regions were compared. The other eight expressed genes flanking the *GIF1 *gene on chromosome 4 show good colinearity to the eight counterparts of the *OsCIN1 *region on chromosome 2 (Figure 1B and Additional file 2). The result indicated that *GIF1 *and *OsCIN1 *rose *via *duplication of a genomic block, which could be as large as 15 Mb (data not shown). As shown in Figure 1A, phylogenetic analysis including cell-wall invertases of *Zea mays*, *Sorghum bicolor*, *Lolium perenne*, *Hordeum vulgare*, *Dendrocalamopsis oldhamii *and *Oryza sativa *showed that *GIF1 *was closer to cell-wall invertases of *Zea mays*, *Hordeum vulgare *and *Dendrocalamopsis oldhamii*, suggesting that this duplication might occur during the genome duplication of grasses [25]. By directly using synonymous substitution rate between the two paralogs (*Ks *= 0.57), and assuming the neutral evolutionary rate of rice genes (~6.5 × 10^-9^substitutions per silent site per year) [26,27], we estimated the time of duplication between *GIF1 *and *OsCIN1 *about 44 million years ago (MYA), a time much earlier than the genus *Oryza *diversified from a common ancestor about 15 MYA [28]. However, this estimated duplication age could be invalid because the regions were likely selected during rice domestication (see below).

To investigate the evidence for functional constraint on both copies at the DNA sequence level, we calculated *Ka *(non-synonymous substitution rate)/*Ks *ratios between *GIF1*, *OsCIN1 *and their homologs in maize, respectively [27]. The respective *Ka*/*Ks *value of *GIF1 *and *OsCIN1 *are 0.275 and 0.168 (p = 4.13E-24, p = 1.92E-50) (Table 1), suggesting strong purifying selection.

**Table 1:** Summary of nonsynonymous (*Ka*) and synonymous (*Ks*) substitutions in *OsCIN1 *and *GIF1*

	Ka	Ks	Ka/Ks	p-value (fister)
GIF1	0.120098	0.436208	0.275322	4.13E-24
OsCIN1	0.124126	0.738313	0.168121	1.92E-50

### Sub-functionalization of *GIF1* and *OsCIN1* by expression differentiation

**Figure 2:**
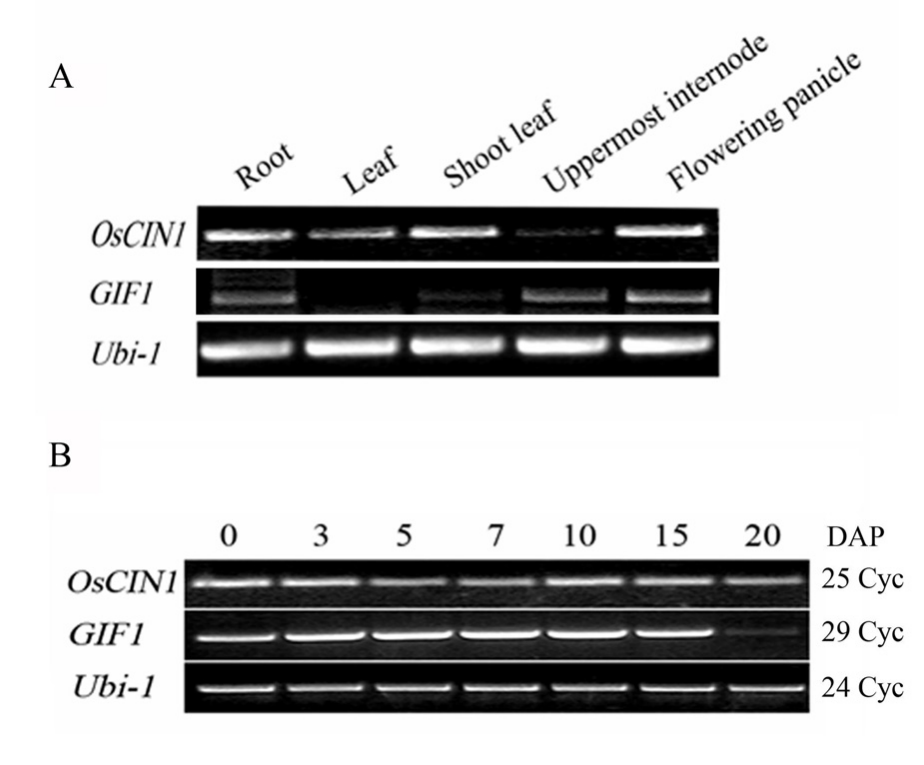
**Different *GIF1 *and *OsCIN1 *expression patterns**. (A) Levels of the *GIF1 *and *OsCIN1 *transcripts were detected by RT-PCR in different tissues. **(B) **Levels of the *GIF1 *and *OsCIN1 *transcripts during grain filling. Note that *OsCIN1 *was constitutively expressed in developing grains. The experiments were repeated twice with similar results, *Ubi-1 *was used as a loading control for RT-PCR. DAP, day after pollination; Cyc, PCR cycles.

Duplicate genes can be maintained by sub-functionalization (the duplicate genes perform different aspects of the original gene's function), or neo-functionalization (one of the genes acquires a novel function), and may facilitate adaptation to environmental change [6-9]. Our previous research has indicated that other *CINs*, including *OsCIN1*, are not functionally redundant to *GIF1 *[22]. Here we further compared the expression patterns of *GIF1 *and *OsCIN1 *in different tissues and grain-filling stages. *GIF1 *transcripts were detected in roots, elongating internodes, shoots and panicles, but not in leaves. In contrast, *OsCIN1 *was expressed strongly in leaves, but weakly in elongating internodes (Figure 2A). During the early grain-filling stage, *OsCIN1 *transcript levels remained high while *GIF1 *transcript levels decreased after 15 days post-pollination (DAP) (Figure 2B). *In situ *hybridization experiments further showed that the *GIF1 *transcript was only detected in the ovular vascular tissue but not in the pericarp and endosperm [22]; in contrast, the *OsCIN1 *transcript was detected in both the pericarp and endosperm [29]. Consistent with the difference in their expression pattern, *GIF1 *was induced in the caryopses supplied with sugars, but *OsCIN1 *was inducible in the leaves treated with sucrose and pathogen [23]. These results evidently showed that *GIF1 *and *OsCIN1 *have differentiated in expression pattern after duplication through altering expression patterns in development and response to environment cues.

### Sub-functionalization of GIF1 and OsCIN1 enzymes

**Figure 3:**
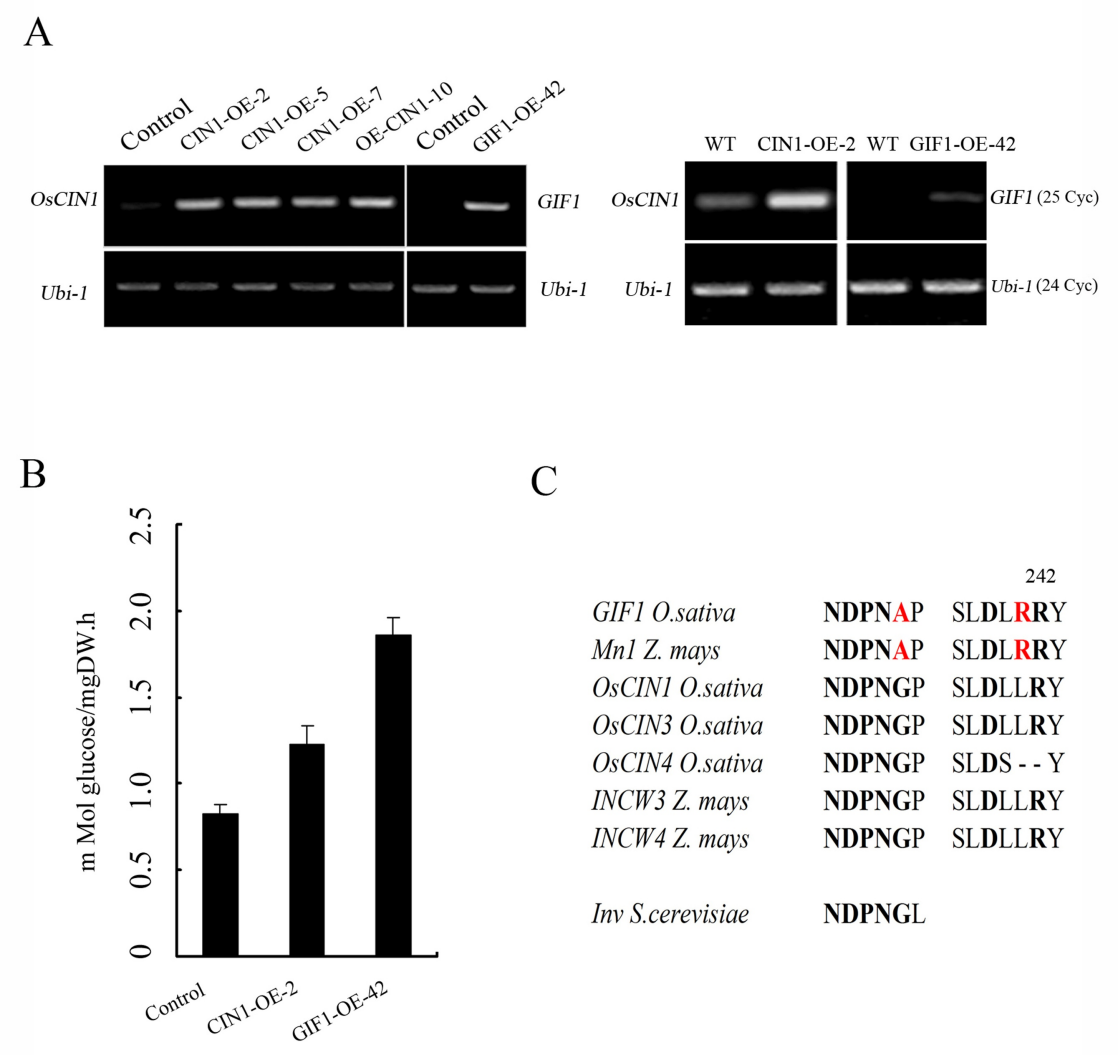
**Enzymatic activity of GIF1 and OsCIN1**. (A) The *GIF1 *and *OsCIN1 *transcript levels in leaves detected by RT-PCR showing *GIF1 *and *OsCIN1 *overexpression in transgenic lines (left). Each one line of GIF1-OE and OsCIN1-OE was analyzed for *GIF1 *and *OsCIN1 *transcript levels respectively, with 25 PCR cycles (right). *Ubi-1*, a loading control for RT-PCR with 24 cycles. Note that line OsCIN1-OE-2 accumulated higher level of *OsCIN1 *transcripts than the level of *GIF1 *transcripts in line GIF1-OE-42. **(B) **CWI activity in leaves of the OsCIN1-OE and GIF1-OE plants and the empty vector control. **C**. Multiple sequence alignment of two conserved regions of cell wall invertases in maize, rice and yeast. The NDPNG domain and Asp-239/Lys-242 are shown in bold. The amino acid difference between GIF1 and OsCIN1 are shown in red color.

Total activity of cell-wall invertases was reduced to 17% of the wild-type in the *gif1 *mutant [22], indicating that GIF1 contributes to the majority of cell-wall invertase activity in early developing grains, although *OsCIN1 *was also expressed at a higher level in developing grains (Figure 2B). In support of this observation, the *OsCIN1 *T-DNA 'knockout' mutant did not show significant defect in grain filling and weight (J. -S. JEON, personal communication). To further determine the functional differentiation of the GIF1 and OsCIN1enzymes, we developed transgenic plants GIF1-OE [22] and CIN1-OE constitutively expressing *GIF1 *and *OsCIN1 *driven by the 35S promoter (Figure 3A). GIF1-OE plants exhibited significantly higher CWI activity than that of CIN1-OE plants although the latter accumulated higher *OsCIN1 *levels (Figure 3A and 3B). These results suggested that GIF1 and OsCIN1 could have different kinetics parameters such as Km and Vmax.

The difference in the kinetics parameters of enzymatic activity might result from the amino acid substitutions, in particular the GIF1 and OsCIN1 proteins contain Ala and Gly residues in the NDPNG domain (motif), respectively (Figure 3C). Phylogenetic reconstruction (Figure 1A and 3C) revealed that the Gly-26-Ala substitution occurred after the *GIF1 *and *OsCIN1 *duplication. The crystal structure of the Arabidopsis CWI indicated that the conserved NDPNG domain is critical for CWI activity [30-32]. However, GIF1 and the homologous maize Mn1 contain a NDPNA motif instead of the NDPNG motif that presents in OsCIN1 and other CWIs, suggesting that the segmental duplication predates maize and rice differentiation. Furthermore, the crystal structure showed that Asp-239 interacted with Lys-242 and both the two amino acids played a crucial role in the transfructosylation process and interacted *via *H-bonds with the bound substrate [32]. A Thr-241-Arg substitution in the Asp-239/Lys-242 region occurred in GIF1 as well as in Mn1. It is noteworthy that the mutation in the *Mn1 *gene also caused shrunk grains [33]. The synteny between the *GIF1 *and *Mn1 *genome regions suggested that they could be orthologues (Additional file 3). These structure differences might contribute to different enzymatic kinetics of the GIF1 and OsCIN1 proteins. Together, these results suggested that *GIF1 *and *OsCIN1 *were subjected to sub-functionalization, or that *GIF1 *(probable *Mn1 *too) might have neofunctionalized, albeit we do not know the ancestral function of the CWIs.

### Different phenotypes of *GIF1-OE* and *CIN1-OE* plants support sub-functionalization of *GIF1* and *OsCIN1*

**Figure 4:**
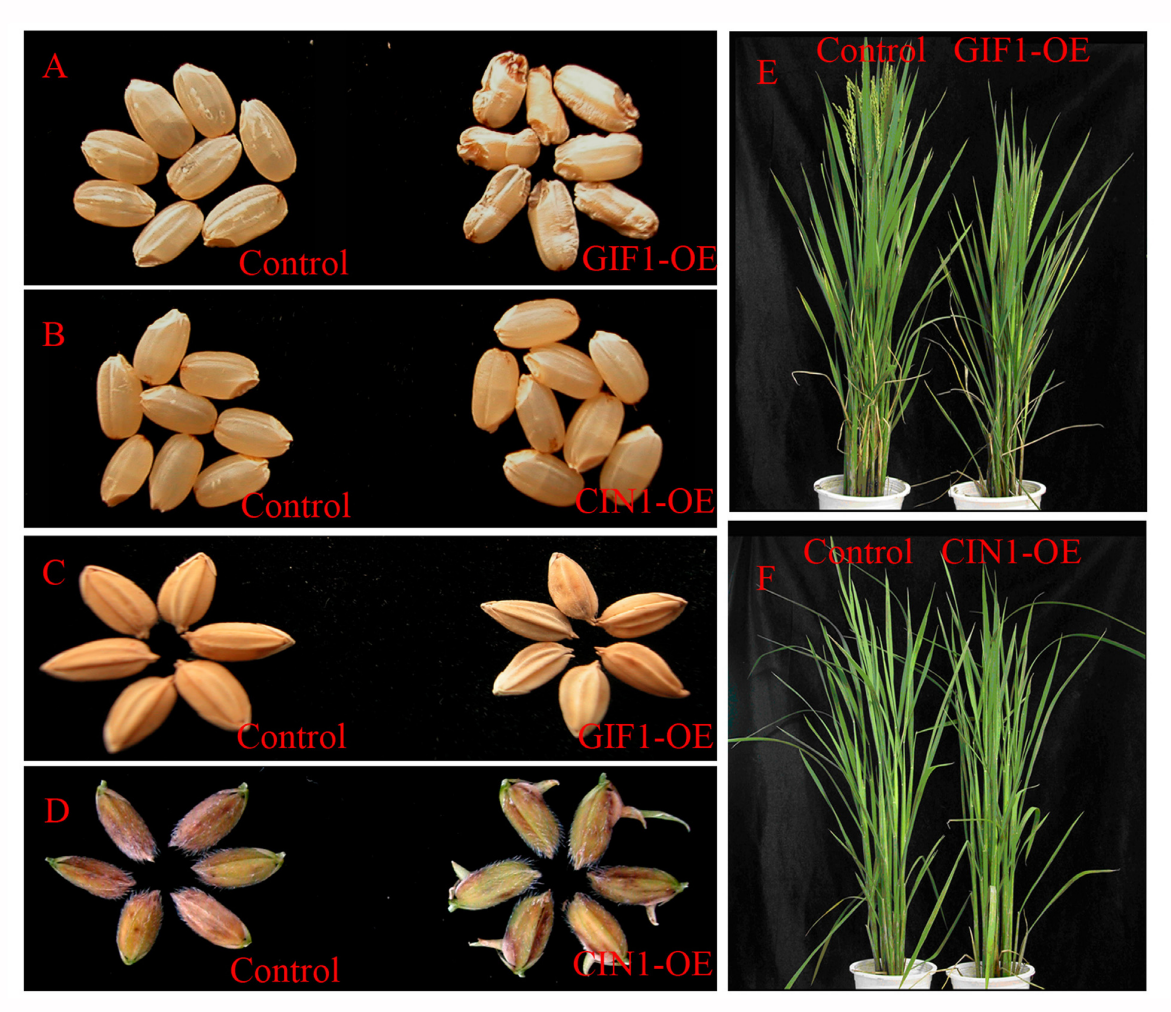
**Phenotypes of OsCIN1-OE and GIF1-OE plants**. (A) The GIF1-OE plants produced badly-filled grains (right), compared to the empty vector control (left). **(B) **The OsCIN1-OE plants produced grains completely filled (right). **(C) **Smaller seeds of the GIF1-OE plants (right), compared to the empty vector control (left). **(D) **The OsCIN1-OE seeds exhibited preharvest sprouting, while the control seeds did not germinate on panicle at the same time. **(E) **The GIF1-OE plants grew dwarfing in comparison with the control. **(F) **The OsCIN1-OE plants were morphologically similar to the control.

To further confirm functional divergence of *GIF1 *and *OsCIN1*, we determined the phenotypes of GIF1-OE and CIN1-OE plants. In addition to producing shrunken grains (Figure 4A and 4C), GIF1-OE plants were also dwarfed in comparison with wild-type plants (Figure 4E). By contrast, CIN1-OE plants did not exhibit any obvious phenotype in grain-filling and plant development (Figure 4B and 4F). Instead, the CIN1-OE seeds exhibited marked preharvest sprouting in 6 of 10 transgenic lines tested, which expressed high *OsCIN1 *levels (Figure 4D and data not shown), a phenomenon never occurring to the wild-type *japonica *control. These observations suggest that *OsCIN1 *might indirectly modulating hormone signaling pathways through interfering sugar metabolism in seed germination, leading to preharvest sprouting, since the sugar regulates rice alpha amylase (34). Together, our results demonstrate that *GIF1 *and *OsCIN1 *have evolved differentially or most likely sub-functionalized after duplication.

### Evidence of *OsCIN1* domestication-selection

**Figure 5:**
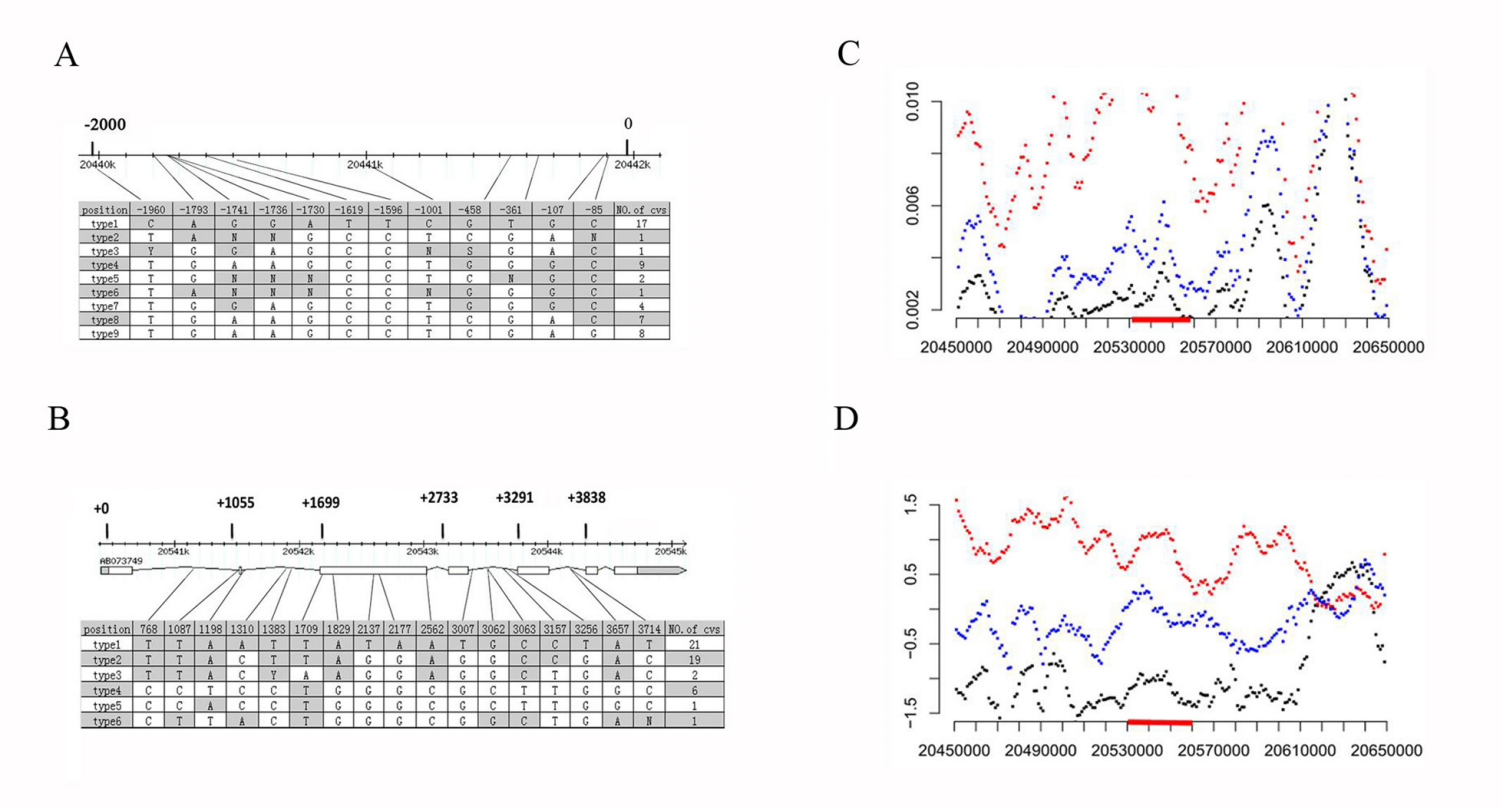
**Nucleotide polymorphisms in *GIF1 *promoters and *OsCIN1 *gene regions**. **(A) **Nucleotide polymorphisms detected in the 2-kb *GIF1 *promoter regions, which are classified into 9 types. The location of *GIF1 *on the chromosome was indicated. **(B) **Nucleotide polymorphisms in the *OsCIN1 *gene regions, which are divided into 6 types. The location of the *OsCIN1 *on the chromosome was indicated. **(C) **The molecular signature of domestication selection of *OsCIN1*. The *OsCIN1 *regions of 25 rice cultivars and 25 wild rice germplasm (Table 2) were sequenced. Haplotype diversity was calculated for nucleotide diversity (π) analysis. **(D) ***Tajima's D-*statistics were calculated with DnaSP version 4.0 for the *OsCIN1 *regions. Sequence positions were indicated with the *OsCIN1 *loci marked red. Red, wild rice; Blue, *indica*; black, *japonica*.

In the previous study, we analyzed artificial selection using the segment sequences of *GIF1*. Here we further analyzed the 2-kb promoter region of *GIF1 *in 25 cultivars and 25 wild rice germplasm (AA genome) (Table 2). We identified nine types of promoter sequences (Figure 5A). According to the promoter sequences, nearly all cultivated rice was classified into type 1, further supporting that the *GIF1 *promoter was artificially selected during rice domestication.

**Table 2:** Cultivars and wild rice germplasm used in this study

Sample name/IRGC no.	Variety name	Origin	Group
8555	DZ78	Bangladesh	*indica*
12883	Mehr	Iran	*indica*
45975	Kalamkati	India	*indica*
32399	Phudugey	Bhutan	*indica*
6307	Jhona 349	India	*indica*
2540	Haginomae Mochi	Japan	*indica*
30416	-	Brazil	*indica*
9177	JC91	India	*indica*
8231	Gie 57	Vietnam	*indica*
9148	TD2	Thailand	*indica*
9060	JC101	India	*japonica*
9062	JC111	India	*japonica*
38994	Bico Branco	Brazil	*japonica*
12793	Kitrana 508	Madagascar	*japonica*
RA4952	Firooz	Iran	*japonica*
66756	Lemont	TX, USA	*japonica*
50448	Canella De Ferro	Brazil	*japonica*
11010	Maintmolotsy 1226	Madagascar	*japonica*
38698	NPE 844	Pakistan	*japonica*
55471	Chodongji	South Korea	*japonica*
27630	Darmali	Nepal	*japonica*
27762	Leung Pratew	Thailand	*japonica*
6513	-	Bangladesh	Southern Asian *indica*
60542	-	Bangladesh	Southern Asian *indica*
31856	-	Bangladesh	Southern Asian *indica*
Dongxiang	-	Dongxiang, China	*O. rufipogon*
Yuan3-9	-	Yunnan, China	*O. rufipogon*
P25	-	Guangdong, China	*O. rufipogon*
P46	-	Hainan, China	*O. rufipogon*
P61	-	Guangxi, China	*O. rufipogon*
80506	-	India	*O. rufipogon*
106505	-	Papua New Guinea	*O. rufipogon*
105426	-	Sri Lanka	*O. rufipogon*
81982	-	India	*O. rufipogon*
81991	-	Myanmar	*O. rufipogon*
105912	-	Thailand	*O. rufipogon*
105958	-	Indonesia	*O. rufipogon*
105960	-	Bangladesh	*O. rufipogon*
106161	-	Laos	*O. rufipogon*
Nepal	-	Nepal	*O. rufipogon*
80470	-	India	*O. nivara*
105705	-	Nepal	*O. nivara*
106345	-	Myanmar	*O. nivara*
105879	-	Bangladesh	*O. nivara*
89215	-	Cambodia	*O. nivara*
106154	-	Laos	*O. nivara*
105784	-	Thailand	*O. nivara*
103407	-	Sri Lanka	*O. nivara*
106105	-	India	*O. nivara*
105327	-	India	*O. nivara*

**Figure 6:**
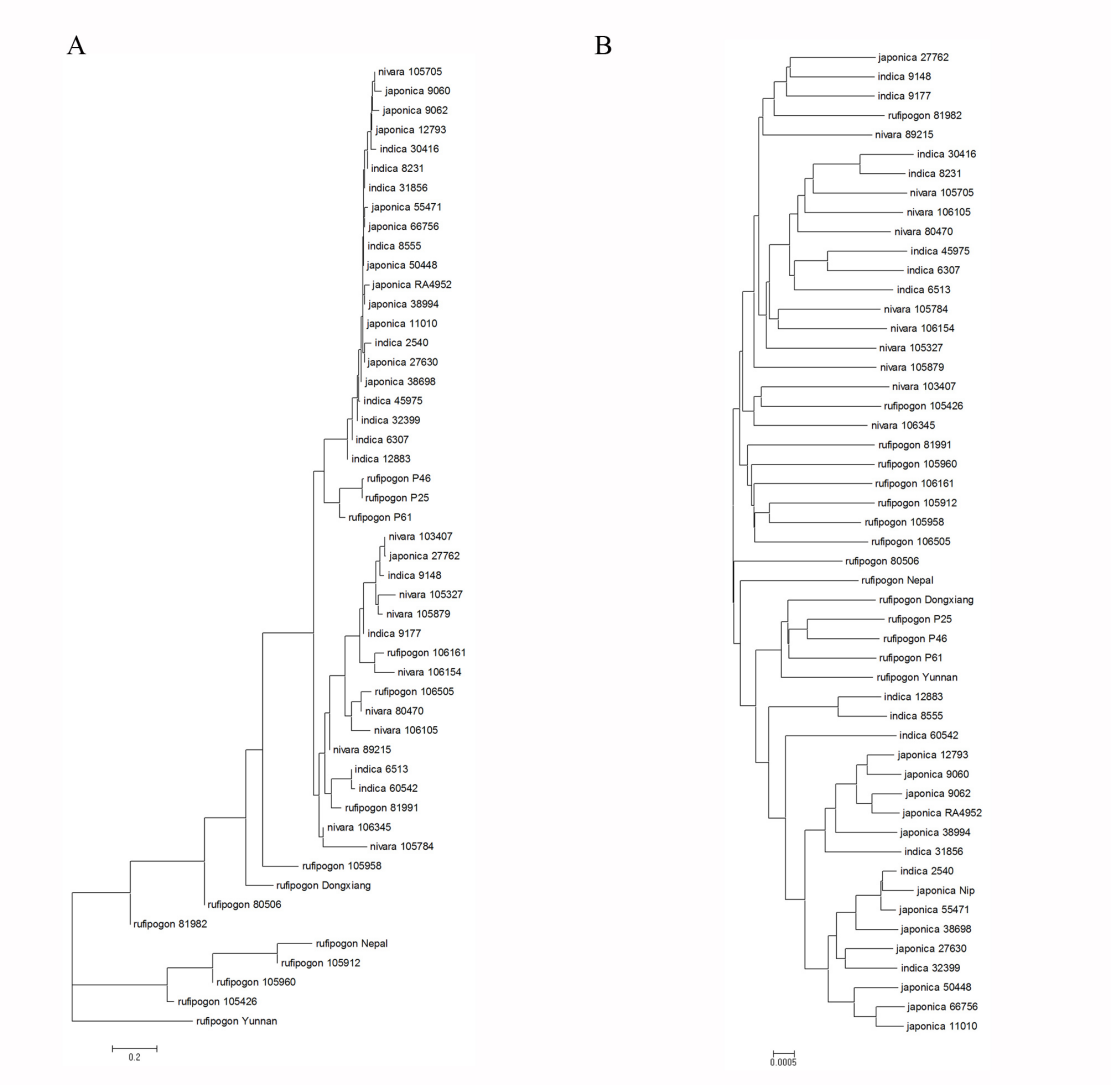
**Phylogenetic trees of rice germplasm**. **(A) **Unrooted neighbor-joining phylogenetic tree of the *OsCIN1 *sequences was established according to sequence divergence of the rice germplasm set (Table 2). **(B) **Unrooted neighbor-joining phylogenetic tree was created according to whole genome SNP in the rice germplasm set (Table 2).

We further sequenced two BACs containing respective *OsCIN1 *and *GIF1 *of the BB-genome of wild rice (*O. punctata*), and found that the coding region of *OsCIN1 *contains more variation than the coding region of *GIF1 *in comparison with the reference AA genome sequences (Additional file 4) [28,35], probably suggesting that *OsCIN1 *and *GIF1 *might have experienced different selection during *Oryza *evolution. To carefully investigate the evolution pattern of the *OsCIN1 *gene, we sequenced the *OsCIN1 *genome regions of the same set of 25 cultivars and 25 wild rice germplasm (Table 2). Results showed that the silent-site nucleotide, θ_π_, of *OsCIN1 *in *japonica *and *indica *were 0.0025 and 0.0038, respectively, lower than θ_π _(0.0097) in wild rice; and also much lower than the genome average θ_π _(0.0052 and 0.0073) in *japonica *and *indica*, respectively (Figure 5C and Additional file 5). Furthermore, the Hudson-Kreitman-Aguade (HKA) test detected a highly significant deviation of *OsCIN1 *from neutrality for cultivated rice compared with the *ADH1 *gene (p = 5.89871E-11) [36], using *O. punctata *as an outgroup (Table 3). The negative deviation in Tajima's D was also consistent with a selective sweep at the *OsCIN1 *locus in both *japonica *and *indica*, but no such a pattern was observed in wild rice (Figure 5D and Additional file 5). These results suggest the *OsCIN1 *gene might also have been artificially selected. We also estimated genetic variation in upstream and downstream regions of *OsCIN1 *in the cultivars and wild rice genomes, and found that the region under selective sweep may extend as long as ~100-Kb. We further constructed a gene tree using 4.6-kb gene regions of *OsCIN1 *from the cultivars and wild rice (Figure 6A). Consequently, all the *japonica *and *indica *accessions formed a cluster in the gene tree, in considerable contrast to a genome tree (Figure 6B) established based on SNP data [37,38], suggesting *OsCIN1 *introgression from one subspecies into another subspecies after domestication-selection, although our data could not rule out the possibility that *OsCIN1 *was extensively selected during rice domestication independently in the two subspecies. All the results strongly support the hypothesis that *OsCIN1 *was selected during rice domestication. However, how the *OsCIN1 *gene has played a role in domestication is still unknown.

**Table 3:** HKA tests of the *OsCIN1 *and *GIF1 *loci^a^

pair	polymorphism site number	sequence length	p value
Outgroup/Cultivar_*OsCIN1*	454	4636	5.90E-11
Cultivar/Cultivar_*OsCIN1*	44	4772	
Outgroup/Cultivar_*GIF1*	441	5980	0.00108
Cultivar/Cultivar_*GIF1*	67	6149	
Outgroup/Cultivar_*ADH1*	170	2573	
Cultivar/Cultivar_*ADH1*	45	2573	

^a^The up-/downstream 2-kb genome regions of OsCIN1 and GIF1 were analyzed for HKA.

To narrow down the selection target in the *OsCIN1 *gene, we examined all the variations in the *OsCIN1 *genome regions, and found that an amino acid substitution (Arg-212-Leu) almost fixed in the rice cultivars, indicating that, unlike *GIF1 *which was selected in the promoter region, this site in the coding region could be the target of artificial selection in the *OsCIN1 *gene (Figure 5B). Further functional characterization of this site will provide more evidence to address how this site has contributed to *OsCIN1 *function in cultivated rice.

## Discussion

### Gene duplication and adaptive conflict

Gene duplication plays a fundamental role in organism evolution by providing genetic materials from which novel functions can arise. Large numbers of duplicate genes were found in genomes, which contributed greatly to the genome structure and function evolution [1-5,39]. In general, the duplicate genes have two fates: first, the duplicate gene lost its function due to chromosome remodeling, deletion, and point mutation (known as non-functionalization); second, the duplicate gene retained for the maintenance of ancestral functions [1-5]. According to adaptive conflict model, adaptive mutations could be prohibited in the case of multifunctional genes, or one mutation that can optimize one function, may compromise the other functions, this mutation will be prohibited [6,40]. The adaptive conflict could be solved by sub-functionalization of duplicate genes. In this case, the duplicate genes would be less constrained and be able to evolve new functions under selection [41,42]. With this scenario, it is common that one gene could have multifunction in nature [40,43].

### Independent selection of *GIF1* and *OsCIN1* mutations

The sequenced genomes of *Oryza sativa*, *Arabidopsis thaliana *and *Populus trichocarpa *all contain a family of cell wall invertases [44-46], some members of these gene families were reported to be involved in growth and development, disease resistance, stress responses and cell death, suggesting that the CWI gene families might have undergone sub- or neo-functionalized in these species.

Through genomic synteny analysis, we showed that *GIF1 *and *OsCIN1 *derived from a segmental duplication from an ancestor, most likely during genome duplication in grass species. After duplication, *GIF1 *and *OsCIN1 *have evolved to gain divergent functions with different expression patterns and enzymatic kinetics parameters through accumulating mutations in cultivated rice. In contrast to *GIF1 *on which domestication selection mainly occurred in the *cis*-regulatory region (Figure 5A), the artificial selection occurred mainly in the coding region of *OsCIN1 *(Figure 5B). Therefore, both *GIF1 *and *OsCIN1 *were most likely subjected to domestication selection, resulting in a cultivated *GIF1 *locus for better harvest, although the biological importance of *OsCIN1 *in domestication remains enigmatic. With this scenario, *GIF1 *and *OsCIN1 *may provide a good genetic model to demonstrate how duplicate genes could evolve and be artificially selected independently during crop domestication with divergent functions derived from accumulation of mutations in the regulatory and coding regions respectively, adding to those systems reported [47,48].

### Differential biological functions of *GIF1* and *OsCIN1*

*GIF1 *is mainly expressed in seed vascular tissues and controls sucrose unloading for starch synthesis at the early grain-filling stage [22]. Overexpression of the *GIF1 *gene produced plants with marked defects both in grain-filling and development, indicating that over-activity of the GIF1 enzyme disrupts sugar homeostasis, a process important to normal grain and plant development. In contrast, OsCIN1 has lower CWI activity compared to GIF1 in the transgenic plants (Figure 3). Consistent with this, no obvious phenotype was observed in CIN1-OE plants except pre-harvest sprouting (Figure 4). Interestingly, *OsCIN1 *might be involved in pathogen defense and stress response [23]. It has been reported that sugars interact with signaling pathways mediated by phytohormones such as GA and ABA during seed germination and seedling development [34,49], which are also involved in stress responses. Preharvest sprouting of the CIN1-OE seeds may implicate a role for *OsCIN1 *in sugar-mediated alpha amylases activation [34]. However, detailed experiments are needed to dissect the *OsCIN1 *function.

## Conclusion

Gene duplication and functional divergence contribute greatly to genetic novelty and adaptive evolution. However, molecular basis of selection and functionalization of duplicate genes remains largely unknown. Based on a set of data including population genetic analysis, fine sequencing of wild rice BACs, phenotyping of transgenic plants and analysis of gene expression and enzymatic activity, we provide a line of evidence that the two rice CWI genes *GIF1 and OsCIN1 *are a pair of duplicate genes and have been subjected to sub-functionalization during evolution or domestication selection. Therefore, duplicate genes could be independently selected towards different functional specificity, either on promoter for different expression pattern or on coding region for different protein function/activity. Our study provides a vivid example for evolution of genetic novelties in a model crop. The interesting phenotype of preharvest sprouting OsCIN1-OE plants suggests that OsCIN1 overaccumulation might disturb sugar balance during seed germination.

## Methods

### Duplication and synteny analysis

The 500-kb radiuses of the *GIF1 *and *OsCIN1 *regions were scanned for homologous pairs. A homolog pair was defined as a single nr-KOME cDNA and its blastn homolog. A total of 18 homologous genes in both sides of the *GIF1 *and *OsCIN1 *loci were compared to establish linearity.

### Sequencing and evolution analysis

To investigate the selective forces acting on *GIF1 *and *OsCIN1 *on the molecular evolution scale, we estimated the statistic *Ka/Ks *using the re-sequencing data (see below) and the maize *Mn1 *and *Incw1-1 *as the outgroup sequence, where *Ka *was the number of nonsynonymous substitutions per nonsynonymous site and *Ks *was the number of synonymous substitutions per synonymous site [27]. *Ka/Ks *values significantly less than 1 were often taken as evidence of constraint. The mean *Ks *of nine pair homolog genes, including *GIF1 *and other eight genes (Additional file 2), in the *GIF1 *and *OsCIN1 *regions were used to estimate the duplication time. Two BAC clones of *O. punctata *(BB genome) from the OMAP project http://www.omap.org/ containing *GIF1 *and *OsCIN1 *respectively, were sequenced.

### Analysis of OsCIN1 and GIF1 domestication

We deeply analyzed the *OsCIN1 *and *GIF1 *sequences from the re-sequenced genomes of 25 rice cultivars and 25 wild rice germplasm (Table 2), which has been done in Dr. Wen Wang's group, using Solexa technology (data not shown). Haplotype diversity was calculated for nucleotide diversity (π), and *Tajima's D- *statistics were calculated with DnaSP version 4.0. The gene tree was created using MEGA software [50]. The sequences then were aligned. The 2-kb up-/downstream genome sequences and the *GIF1*, *OsCIN1 *coding sequences were used for HKA test as described [36]. Sequences from wild rice *O. punctata *(BB genome) from the OMAP project http://www.omap.org/ were used as outgroups for the HKA test. The DNA phylogenetic tree was constructed by neighbor-joining method using MEGA. The known or predicted CWI genes with high sequence similarity to *GIF1 *from *Oryza sativa*, *Lolium perenne*, *Hordeum vulgare*, *Dendrocalamopsis oldhamii *and the recently released *Zea mays *and *Sorghum bicolor *genomes were used in this study.

### Development and growth of *OsCIN1-OE* transgenic plants

The full-length *OsCIN1 *coding sequence was PCR-amplified from ZH11 cDNA by using the primers 5'-TCTAGTACAAAACAATGGGGACTC-3' and 5'-CGGAAAACCTCTTTATTATCTGTA-3'. The amplified fragment was subsequently cloned into the vector 35S-C1301 and transformed into ZH11 to generate 25 independent ectopic expression lines as described [22]. All transgenic materials were assayed in the second (T1) or third (T2) generations with 10-24 sibling plants grown in the paddy field to ensure agronomic traits.

### Invertase activity assay

The caryopses were ground in the extraction buffer, and the extraction was centrifuged at 12,000 g for 10 min. The pellet was washed twice then re-suspended in the extraction buffer. Insoluble invertase activity was assayed as described [22].

### RNA preparation and analysis

Total RNA was prepared from rice tissues using TRIzol reagent according to the manufacture's protocol (GIBCO BRL). For RT-PCR, 1-5 ug total RNA was used for the first-strand cDNA synthesis with the SuperScript III System (Invitrogen). RT-PCR analysis of *GIF1 *and *OsCIN1 *was performed with the primers [22,23].

### Accession numbers

All sequences have been deposited in GenBank under accession numbers GU797900-GU798049.

## Authors' contributions

EW, SG, B-RL, WW and ZH designed research. EW, LZ, HZ, LL, QW and QL performed research. EW, XX, SG, B-RL, WW and ZH analyzed data. EW, SG, B-RL, WW and ZH wrote the paper. All authors read and approved the final manuscript.

## Supplementary Material

Additional file 1 Table S1. Summary of the number of amino acid substitutions per site of eight cell wall invertases. Click here for file

Additional file 2 Table S2. Genes and positions on the *GIF1* and *OsCIN1* chromosome regions. Click here for file

Additional file 3 **Figures S1. The synteny between the rice *GIF1* genome regions (chromosome 4) and the maize *Mn1* genome regions (chromosome 10)**. High linearity indicates their duplication from the same ancestor fragment(s). Click here for file

Additional file 4 **Figure S2. Sequence comparison of the *GIF1* and *OsCIN1* coding regions in japonica (O. sativa) and O. punctata (BB genome)**. Two BAC clones containing respective *OsCIN1 *and *GIF1 *of the BB-genome wild rice (*O. punctata*) were sequenced. **A**. Sequence alignment of the *GIF1 *coding regions in *japonica *(*O. sativa*) and *O. punctata*. **B**. Sequence alignment of the *OsCIN1 *coding regions in *japonica *(*O. sativa*) and *O. punctata*. Note that the *OsCIN1 *sequence has more divergence than *GIF1*. Click here for file

Additional file 5 Table S3. Nucleotide polymorphisms and neutrality test for domestication signature of OsCIN1. Click here for file
