# Supplementary material for: Duplication and independent selection of cell-wall invertase genes GIF1 and OsCIN1 during rice evolution and domestication
Source: BMC Evol Biol. 2010 Apr 23;10:108. doi: 10.1186/1471-2148-10-108 (PMC2873416; doi:10.1186/1471-2148-10-108)
Supplement: Additional file 1 — Table S1. Summary of the number of amino acid substitutions per site of eight cell wall invertases. [file 1471-2148-10-108-S1.PDF]

Table S1: Summary of the number of amino acid substitutions per site of eight cell wall invertases

	OsCIN1	*GIF1*	*OsCIN3*	*OsCIN4*	*OsCIN5*	*OsCIN6*	*OsCIN7*	*OsCIN8*
OsCIN1								
GIF1	0.274							
OsCIN3	0.400	0.425						
OsCIN4	0.587	0.612	0.648					
OsCIN5	0.658	0.669	0.701	0.622				
OsCIN6	0.664	0.674	0.696	0.627	0.380			
OsCIN7	0.669	0.685	0.685	0.597	0.345	0.285		
OsCIN8	0.653	0.690	0.701	0.612	0.345	0.292	0.059	

The number of amino acid substitutions per site from analysis between sequences is shown. All results are based on the pairwise analysis of the CWI sequences. Analyses were conducted using the Poisson Correction Method in MEGA4. All positions containing gaps and missing data were eliminated from the dataset (Complete deletion option).
