# Supplementary material for: Duplication and independent selection of cell-wall invertase genes GIF1 and OsCIN1 during rice evolution and domestication
Source: BMC Evol Biol. 2010 Apr 23;10:108. doi: 10.1186/1471-2148-10-108 (PMC2873416; doi:10.1186/1471-2148-10-108)
Supplement: Additional file 2 — Table S2. Genes and positions on the GIF1 and OsCIN1 chromosome regions. [file 1471-2148-10-108-S2.PDF]

Table S2: Genes and positions on the *GIF1* and *OsCIN1* chromosome regions

Gene	cDNA	Chromosome position	Gene	cDNA	Chromosome position	*Ks*
Os04g33200	AK107531	19737863 bp - 19745773	*Os02g32504*	AK071231	19170490 bp - 19174182	0.58748
Os04g33450	AK121434	19891562 bp - 19893402	*Os02g32650*	AK063839	19297688 bp - 19300007	0.77613
Os04g33590	AK102032	19993750 bp - 19999824	*Os02g32970*	AK119210	19536399 bp - 19540643	0.55918
Os04g33640	AK121151	20027785 bp - 20032831	*Os02g33000*	AK073663	19564355 bp - 19568327	0.63916
GIF1	AK072276	20077044 bp - 20081886	***OsCIN1***	AK121403	19627655 bp - 19632362	0.11741
Os04g33750	AK121192	20087349 bp - 20090652	*Os02g33140*	AK101459	19655451 bp - 19658581	0.78715
Os04g33860	AK108662	20163494 bp - 20164440	*Os02g33330*	AK073251	19742901 bp - 19743831	0.56973
Os04g33950	AK067581	20204822 bp - 20210178	*Os02g33430*	AK068689	19813312 bp - 19818420	0.45787
Os04g33990	AK068271	20234257 bp - 20235407	*Os02g33550*	AK065946	19899495 bp - 19900773	0.66965
