# Supplementary figures and images for: Duplication and independent selection of cell-wall invertase genes GIF1 and OsCIN1 during rice evolution and domestication
Source: BMC Evol Biol. 2010 Apr 23;10:108. doi: 10.1186/1471-2148-10-108 (PMC2873416; doi:10.1186/1471-2148-10-108)

Oryza sativa japonica chromosome 4

Mnl

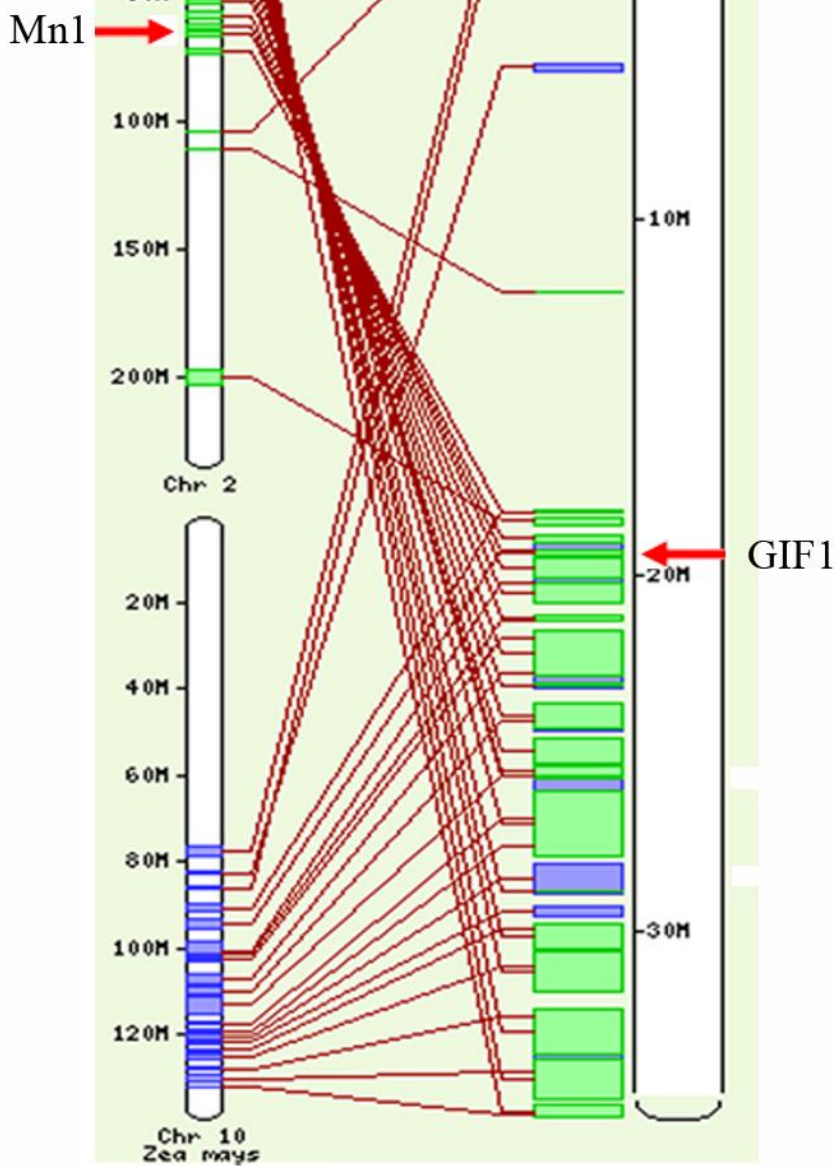

Supplement: Additional file 3 — Figures S1. The synteny between the rice GIF1 genome regions (chromosome 4) and the maize Mn1 genome regions (chromosome 10). High linearity indicates their duplication from the same ancestor fragment(s). [file 1471-2148-10-108-S3.PDF]
