# Supplementary material for: Duplication and independent selection of cell-wall invertase genes GIF1 and OsCIN1 during rice evolution and domestication
Source: BMC Evol Biol. 2010 Apr 23;10:108. doi: 10.1186/1471-2148-10-108 (PMC2873416; doi:10.1186/1471-2148-10-108)
Supplement: Additional file 4 — Figure S2. Sequence comparison of the GIF1 and OsCIN1 coding regions in japonica (O. sativa) and O. punctata (BB genome). Two BAC clones containing respective OsCIN1 and GIF1 of the BB-genome wild rice (O. punctata) were sequenced. A. Sequence alignment of the GIF1 coding regions in japonica (O. sativa) and O. punctata. B. Sequence alignment of the OsCIN1 coding regions in japonica (O. sativa) and O. punctata. Note that the OsCIN1 sequence has more divergence than GIF1. [file 1471-2148-10-108-S4.PDF]

A

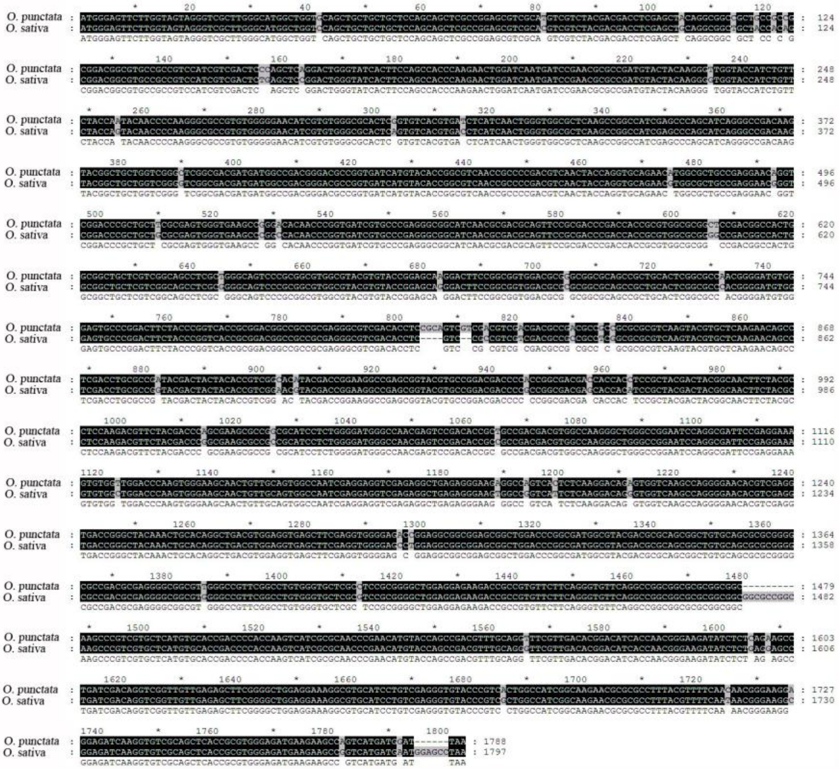

B

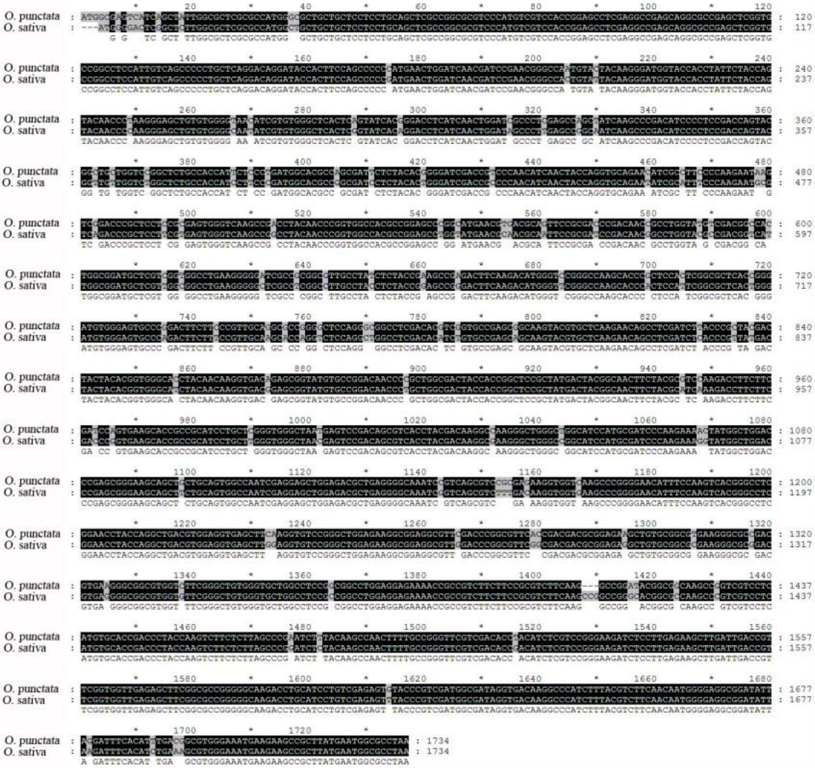

Figure S1

Sequence comparison of the *GIF1* and *OsCIN1* coding regions in *japonica* (*O. sativa*) and *O. punctata* (BB genome). Two BAC clones containing respective *OsCIN1* and *GIF1* of the BB-genome wild rice (*O. punctata*) were sequenced. **(A)** Sequence alignment of the *GIF1* coding regions in *japonica* (*O. sativa*) and *O. punctata*. **(B)** Sequence alignment of the *OsCIN1* coding regions in *japonica* (*O. sativa*) and *O. punctata*. Note that the *OsCIN1* sequence has more divergence than *GIF1*.
